# Impact of early surgical complications on kidney transplant outcomes

**DOI:** 10.1186/s12893-024-02463-7

**Published:** 2024-05-27

**Authors:** Michelle Minkovich, Nikita Gupta, Michelle Liu, Olusegun Famure, Yanhong Li, Markus Selzner, Jason Y. Lee, S. Joseph Kim, Anand Ghanekar

**Affiliations:** 1 Kidney Transplant Program, Toronto General Hospital, University Health Network, 585 University Avenue, 9-MaRS-9050, Toronto, ON M5G 2N2 Canada; 2 Division of Urology, University Health Network, Toronto, Canada; 3 Division of Nephrology, University Health Network, Toronto, Canada; 4 Department of Medicine, University of Toronto, Toronto, Canada; 5 Division of General Surgery, University Health Network, Toronto, Canada; 6 Department of Surgery, University of Toronto, Toronto, Canada

**Keywords:** Surgical complications, Kidney, Transplantation, Clinical outcomes

## Abstract

**Background:**

Kidney transplantation (KT) improves clinical outcomes of patients with end stage renal disease. Little has been reported on the impact of early post-operative surgical complications (SC) on long-term clinical outcomes following KT. We sought to determine the impact of vascular complications, urological complications, surgical site complications, and peri-graft collections within 30 days of transplantation on patient survival, graft function, and hospital readmissions.

**Methods:**

We conducted a single-centre, observational cohort study examining adult patients (≥ 18 years) who received a kidney transplant from living and deceased donors between January 1st, 2005 and December 31st, 2015 with follow-up until December 31^st^, 2016 (*n* = 1,334). Univariable and multivariable analyses were performed with Cox proportional hazards models to analyze the outcomes of SC in the early post-operative period after KT.

**Results:**

The cumulative probability of SC within 30 days of transplant was 25%, the most common SC being peri-graft collections (66.8%). Multivariable analyses showed significant relationships between Clavien Grade 1 SC and death with graft function (HR 1.78 [95% CI: 1.11, 2.86]), and between Clavien Grades 3 to 4 and hospital readmissions (HR 1.95 [95% CI: 1.37, 2.77]).

**Conclusions:**

Early SC following KT are common and have a significant influence on long-term patient outcomes.

**Supplementary Information:**

The online version contains supplementary material available at 10.1186/s12893-024-02463-7.

## Introduction

Kidney transplantation (KT) is the treatment of choice for patients with end-stage renal disease, offering improved survival and quality of life for the vast majority of patients when compared to other renal replacement therapies [1]. Despite improving patient and graft survival rates over time, the morbidity and mortality associated with postoperative complications remain a significant clinical concern [1].

Early postoperative surgical complications (SC) vary in severity and can be classified according to Clavien Grades [2, 3]. The main categories of SC associated with KT are vascular, urological, peri-graft fluid collections, and surgical site complications. Vascular complications are often the most serious and urgent category of SC, primarily consisting of renal artery stenosis, renal artery thrombosis, pulmonary embolism, deep vein thrombosis, and peri-graft hemorrhage. Vascular complications are reported to range in incidence from 2.5% [4] to 13.5% [5] and are associated with reduced 5-year patient and graft survival [6].

Post-transplant urological complications include urinary leaks and collecting system obstructions [7–9], as well as ureteric calculi and bladder stones [10]. The incidence of urological complications ranges from 3 to 20% [7, 9, 11–15], and is associated with morbidity, graft impairment, graft loss, and recipient death [9].

Surgical site complications include wound infections, wound dehiscence, and abscesses. In the literature, the incidence of surgical site complications ranges from 2% [16] to 26% [14]. One Canadian study examined surgical site complications with a focus on infections, reporting an incidence rate of 8% [17]. Surgical site complications have been associated with decreased graft survival [18] as well as recipient death [19]. Moreover, 2.5% of post-transplant rehospitalizations have been reported to be caused by surgical site infections, and patients with these infections are more likely to require a second operation compared to those without surgical site infections [20].

While many studies have investigated the relationship between specific types of SC and post-transplant outcomes, no studies have examined SC in aggregate. Further limitations of existing literature include heterogeneity in the definitions and documentation of SC, as well as variation among centres in the management of SC. Finally, few studies have examined differences in transplant outcomes based on a standardized classification system for the severity of SC.

The goal of this study was to utilize well-defined criteria to determine the incidence and severity of SC in a large single-centre cohort of KT patients and to investigate their overall impact on clinical outcomes.

## Methods

### Study design and population

A single-centre observational cohort study was conducted on adult kidney transplant recipients (≥ 18 years of age) with living and deceased donors, who were transplanted between January 1st, 2005 and December 31st, 2015, with follow-up until December 31^st^, 2016. Kidney transplant candidates were evaluated and listed in accordance with the Adult Kidney Transplant Listing Guidelines as published by the Ministry of Health in the province of Ontario, Canada [21]. To be eligible for active listing, transplant candidates required a minimum hemoglobin of 90 g/dL. Iliac vessels were evaluated with Doppler ultrasound and candidates with moderate or severe iliac plaques/calcification or blunted arterial waveforms were further evaluated with CT scan to confirm the presence of healthy areas for clamping of the vessels and graft renal artery anastomosis. Recipient candidates with BMI > 35 were evaluated on a case-by-case basis for body weight distribution, abdominal pannus, and technical feasibility of accessing the iliac fossa for kidney transplantation. Determination of candidate suitability for listing was independent of living donor availability. Desensitization for ABO or HLA antibodies was not performed in this cohort.

Deceased donor kidneys were evaluated and allocated in accordance with published provincial guidelines [22]. Horseshoe kidneys and pediatric en bloc donor kidneys were not utilized. Living donor kidneys were utilized in accordance with Canadian national guidelines [23, 24]. Multiple renal arteries, short vessels, or those requiring backbench reconstruction were not considered contraindications.

Patients were excluded from the study if they had prior or simultaneous non-kidney transplants, their transplants done at an outside centre, or they had a prior history of KT.

### Data sources

Data sources included electronic medical records from our institution’s Organ Transplant Tracking Record and Electronic Patient Record systems, as well as data from the Comprehensive Renal Transplant Research Information System [25, 26]. This study received approval from our institution’s research ethics board. The data were independently collected and audited by multiple research personnel. Research personnel abstracted data from text documents such as discharge summaries, follow-up clinic notes, progress notes, and relevant diagnostic and test results (such as Doppler ultrasound reports). Uncertain SC cases were adjudicated by clinical experts.

### Definition of surgical complications

Graft-related SC data was collected and coded using the International Statistical Classification of Diseases and Related Health Problems -10^th^ edition [27] and were grouped into four major categories: vascular complications, urological complications, surgical site complications, and peri-graft fluid collections. Vascular complications included renal artery/vein stenosis and thrombosis, which were only considered SC if they required intervention [28, 29]. Peri-graft fluid collections were subcategorized into lymphoceles (which also included any non-specific fluid collections) and hematomas. Hematomas were defined as a drop in hemoglobin ≥ 20 g/L over a 24-h period within 21 days of transplant, with an ultrasound indicating a significant collection. Urological complications included ureteral strictures, urinary obstructions, and urinary leaks, based on Doppler ultrasound reports and clinic notes. Finally, surgical site complications included wound infections, transplant wound dehiscence, and abscesses, based on discharge summaries, clinic notes, and progress notes. Early SC were defined as any SCs that occurred within 30-days post-transplant.

### Statistical analysis

SC were first analyzed as an outcome variable. Descriptive statistics were used to determine the incidence and trends of SC in the study population, and to summarize recipient, donor, and transplant baseline characteristics. For descriptive statistics, normally distributed continuous variables were presented as mean (± standard deviation, SD). Student’s t-tests were used to compare the difference between groups (patients with SC versus those without). Skewed distributed continuous variables were presented as median (interquartile range) and comparisons were made using the Wilcoxon Rank Sum Test. Categorical variables were reported as frequencies and percentages and comparisons were made using the Chi-squared test.

The incidence proportion of SC within 30 days post-transplant was calculated as the percentage of patients with surgical complications out of the total sample size. Kaplan–Meier (KM) estimators were used to estimate the cumulative probability that study subjects remained SC-free after transplant. The log-rank test was used to assess the difference in survival functions between study subjects with and without SC.

SCs were then analyzed as an exposure variable in its association with clinical outcomes, on the condition that KTR survived with a functioning graft for at least 30 days post-transplant. Post-transplant outcomes of interest included death-censored graft failure, death with graft function, total graft failure (defined as a composite of the first two outcomes), estimated glomerular filtration rate (eGFR), and hospital readmissions post-transplant (defined as at least one overnight stay in hospital). eGFR and hospital readmissions were analyzed over the first year post-transplant, whereas all other outcomes were followed up until the study end date (December 31^st^, 2016). SCs were examined as a risk factor in two ways: its presence or absence, and according to the severity of the complications using Clavien Grades 1 to 5. The Clavien score assesses the severity of a complication by the intervention required to address it [2] (Supplementary index, Table 1).

To examine the effects of SC on transplant outcomes, the Kaplan Meier product limit method was used to estimate the cumulative probabilities of death with graft function, death-censored graft failure, total graft failure, and hospital readmissions within one-year post-transplant. For both univariable and multivariable analyses, Cox proportional hazard models were used to analyze the effect of early SC on post-transplant clinical outcomes. Covariates for the multivariable models included recipient variables: age, sex, race, BMI, time on dialysis, peak PRA, history of diabetes; donor variables: age, expanded criteria status, BMI, DCD status; and transplant characteristics: delayed graft function, cold ischemic time, induction type, and transplant era. Violations of the proportionality assumption were checked using log(-log(S(t))) plots, interactions between risk factors and time, as well as Schoenfeld residuals. Outcomes were stratified based on the absence versus presence of SC, as well as by severity of SC.

An additional sensitivity analysis was conducted to examine the effects of SC on clinical outcomes, separated by time of SC occurrence (1 and 2 weeks post KT). Multivariable Cox proportional hazard models were used for this sensitivity analysis. Missing values were handled using multiple imputation, accounting for the uncertainties when predicted missing values, both within and between imputed datasets.

All analyses were performed using Stata/MP, version 12.0 [30]. A two-tailed *p*-value of < 0.05 was deemed statistically significant.

## Results

### Study population

**Fig. 1:**
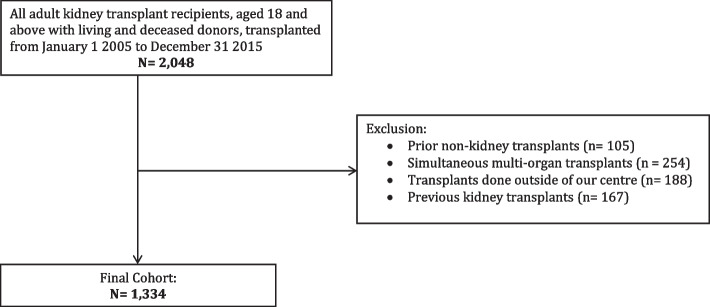
Population flow diagram for study cohort

After applying the inclusion and exclusion criteria, the final study cohort comprised of 1,334 kidney transplant recipients (Fig. 1). As shown in Table 1, 60.3% of recipients were males and 60.6% were white. The mean recipient age at transplantation was 51.3 ± 13.5 years. Slightly over half of transplants (52.0%) were from deceased donors, of which 18.7% were from expanded criteria donors (ECD).

**Table 1:** Baseline characteristics of study population at time of transplant

Variables	Whole cohort (*n* = 1334)		Any graft-related surgical complications within one 30 days post-transplant		
	**Number of Patients (% with surgical complications)**	**Characteristics**	**Yes (*n* = 329)**	**No (*n* = 1005)**	***P*-value**
**Recipient age at transplant (years)**	1334 (24.7%)	51.3 (± 13.5)	53.3 (± 13.0)	50.7 (± 13.6)	0.002
**Recipient sex**					
Male	798 (25.7%)	60.3%	63.6%	59.2%	0.16
**Recipient race**					
Non-white	478 (26.1%)	39.4%	43.0%	38.2%	0.15
White	736 (22.5%)	60.6%	57.0%	61.8%	
**Recipient BMI (kg/m**^**2**^**)**	1285 (24.8%)	27.2 (± 5.6)	27.4 (± 5.7)	27.1 (± 5.5)	0.56
**Donation after cardiac death (DCD)**					
**No**	1185 (24.2%)	88.8%	87.2%	89.4%	0.29
**Yes**	149 (28.2%)	11.2%	12.8%	10.7%	
**Time on dialysis (years)**	1334 (24.7%)	3.2 (1.2, 5.6)	3.5 (1.8, 5.8)	3.0 (1.0, 5.5)	0.01
**Peak PRA**					
= 0%	659 (22.0%)	49.9%	45.5%	51.3%	0.07
> 0%	662 (24.8%)	50.1%	54.6%	48.7%	
**Recipient history of diabetes mellitus**					
Yes	422 (27.7%)	31.9%	36.7%	30.4%	0.04
**Donor age at donation (years)**	1332 (24.7%)	47.6 (± 14.5)	49.8 (± 13.6)	46.9 (± 14.7)	0.001
**Donor type**					
Living	640 (22.2%)	48.0%	43.2%	49.6%	0.04
Deceased	694 (26.9%)	52.0%	56.8%	50.5%	
**Expanded criteria donors**					
Yes	249 (32.5%)	18.7%	16.7%	24.6%	0.001
**Double kidney**					
Yes	46 (34.8%)	3.5%	5.0%	3.0%	0.08
**Number of renal arteries**					
1	1043 (23.9%)	80.2%	79.8%	80.4%	0.83
2 or more	257 (24.5%)	19.8%	20.2%	19.6%	
**Number of renal veins**					
1	1215 (24.3%)	93.7%	94.9%	93.3%	0.33
2 or more	82 (19.5%)	6.3%	5.1%	6.7%	
**Cold ischemic time (hours) (deceased only)**	644 (26.6%)	11.0 (8.0, 15.1)	11.9 (8.8, 16.4)	10.8 (7.9, 14.2)	0.01
**Type of induction**					
Non-depleting agent	350 (19.1%)	26.2%	20.4%	28.2%	< 0.001
Depleting agent	962 (25.8%)	72.1%	75.4%	71.0%	
No Induction	22 (63.6%)	1.7%	4.3%	0.8%	
**Transplant era**					
2005–2009	537 (28.9%)	40.3%	47.1%	38.0%	0.01
2010–2012	386 (21.0%)	28.9%	24.6%	30.4%	
2013–2015	411 (22.6%)	30.8%	28.3%	31.6%	

Percentages in the “Number of patients (% with surgical complications) are row percentages (for example, 25.7% of males in the study population had a surgical complication. Percentages in the other columns are column percentages (for example, of those with surgical complications, 63.6% were males)

Factors significantly different between patients with and without SC included older recipient age, longer recipient time on dialysis, recipient history of diabetes, older donor age, deceased donor grafts, longer cold ischemic time, induction therapy with a depleting agent, and transplant era.

Similar findings emerged from sub-analyses of living donor and deceased donor transplant recipients, as well as from analyses of recipients grouped by Clavien Grade of SC (Supplementary Tables S2a and S2b).

### Incidence of surgical complications

As shown in Table 2, 329 (24.7%) of the 1,334 patients in the final cohort had one or more SC during the first 30 days after transplantation, with 13% of all first SC occurring during the first week. When stratified by donor type, the incidence rate for any first SC was significantly higher for ECD transplants (Supplementary Appendix, Figures S1a and S1b). There was a trend of decreasing SC incidence in the more recent transplant years (Supplementary Appendix, Figure S2).

**Table 2:** Descriptive distribution of surgical complications within 30 days post-transplant

Overall graft-related surgical complications	Number of transplants	Within 30 days post-transplant			
		**Type of Graft-Related Surgical Complication**	**Number of Transplants with Graft-Related Surgical Complications**	**Sub-Type of Graft-Related Surgical Complications**	**Number of Sub-Type Graft-Related Surgical Complications**^**a**^
**At least one graft-related surgical complication**	329	Vascular Complications	19 (5.1%)	Renal Artery/Vein Stenosis	2
				Renal Artery/Vein Thrombosis	19
				Total	21
		Peri-graft Fluid Collections	248 (66.8%)	Lymphocele	100
				Hematoma	177
				Total	277
		Urological Complications	41 (11.1%)	Ureteral Stricture & Urinary Obstruction	35
				Urinary Leak	6
				Total	41
		Surgical Site Complications	63 (17.0%)	Total	63
**No graft-related surgical complications**	1005				
**Total**	1334		371		402^a^

^a^A given patient can have more than 1 SC

### Descriptive distribution of surgical complications

Of the total number of SC that patients experienced, 248 (66.8%) were peri-graft fluid collections, 63 (17%) were surgical site complications, 41 (11.1%) were urological complications, and 19 (5.1%) were vascular complications. The most common peri-graft fluid collections were hematomas; the most common urological complications were ureteral strictures and urinary obstruction; and the most common vascular complications were renal artery/vein thrombosis. Surgical site complications were categorized under wound infections.

### Clinical outcomes of surgical complications: SC as an exposure variable

The final sample size for clinical outcomes analysis was 1,303 patients, with 17 patients excluded from the original cohort due to graft failure, death, or loss to follow-up within 30 days post-transplant, 10 of which had an SC (Supplementary Appendix, Table S4). Univariable and multivariable analyses were performed following two methods of SC categorization: (1) the presence or absence of complications and (2) by severity (Clavien Grades). There were no Clavien Grade 5 SC cases in our study sample.

**Fig. 2:**
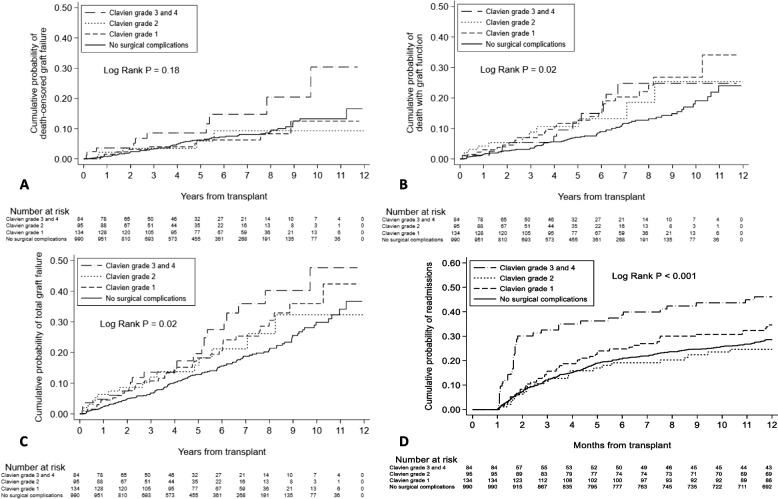
Cumulative probability of: **a** death censored graft failure; **b** death with graft function; **c** total graft failure and **d**) hospital readmissions, stratified by severity of graft-related surgical complication

The cumulative probabilities of different clinical outcomes, separated by severity of SC, are depicted in Kaplan–Meier plots (Fig. 2). In univariable (unadjusted) Cox proportional hazards models, presence of any SC was associated with a significantly higher probability of death with graft function (HR 1.75 [95% CI: 1.23, 2.50]), total graft failure (HR 1.52 [95% CI: 1.14, 2.01]) and hospital readmissions (HR 1.30 [95% C.I.: 1.04, 1.62]), but without a statistically significant association with death censored graft failure.

Multivariable analyses showed no significant relationship between having SC and any of the clinical outcomes of interest (death censored graft failure, death with graft function, total graft failure, and hospital readmissions) (Table 3). However, when exposure was stratified by Clavien Grades, having Grade 1 SC was significantly associated with death with graft function (HR 1.78 [95% CI: 1.11, 2.86]). In addition, having a Grade 3 to 4 SC was significantly associated with hospital readmissions (HR 1.95 [95% CI: 1.37, 2.77]).

**Table 3:** Cox proportional hazards model for effect of surgical complications within 30 days post-transplant on outcomes

Risk factors	Transplant outcomes (univariable models)				Transplant outcomes (multivariable models)			
	**Death-Censored Graft Failure**	**Death with Graft Function**	**Total Graft Failure**	**Hospital Readmissions**	**Death-Censored Graft Failure**	**Death with Graft Function**	**Total Graft Failure**	**Hospital Readmissions**
	Hazard Ratio (95% C.I.)	Hazard Ratio (95% C.I.)	Hazard Ratio (95% C.I.)	Hazard Ratio (95% C.I.)	Hazard Ratio (95% C.I.)	Hazard Ratio (95% C.I.)	Hazard Ratio (95% C.I.)	Hazard Ratio (95% C.I.)
**Any surgical complications (Yes vs. No)**	1.20 (0.75, 1.92)	1.75 (1.23, 2.50)	1.52 (1.14, 2.01)	1.30 (1.04, 1.62)	0.93 (0.56, 1.55)	1.30 (0.89, 1.91)	1.18 (0.87, 1.59)	1.24 (0.98, 1.56)
**By Clavien Grade:**								
Grade One vs. No	0.90 (0.45, 1.81)	1.81 (1.16, 2.83)	1.43 (0.98, 2.07)	1.25 (0.92, 1.71)	0.65 (0.31, 1.36)	1.78 (1.11, 2.86)	1.25 (0.84, 1.86)	1.18 (0.86, 1.64)
Grade Two vs. No	1.00 (0.40, 2.48)	1.71 (0.91, 3.21)	1.40 (0.84, 2.35)	0.85 (0.55, 1.30)	0.78 (0.30, 1.99)	1.17 (0.61, 2.22)	0.97 (0.57, 1.65)	0.83 (0.54, 1.28)
Grade Three and Four vs. No	2.03 (1.04, 3.95)	1.66 (0.88, 3.11)	1.81 (1.15, 2.87)	2.04 (1.45, 2.86)	1.79 (0.88, 3.63)	0.85 (0.43, 1.68)	1.27 (0.79, 2.06)	1.95 (1.37, 2.77)

Multivariable analyses did not demonstrate significant relationships between having any SC and reduced eGFR at one-year after KT or between SC of particular Clavien Grades and reduced eGFR at the one-year mark. However, there was a trend towards a significant reduction in eGFR at one-year post-transplant for patients with SC of Clavien Grade 3 or above (Supplementary Appendix, Figure S3).

A sensitivity analysis investigating the effects of SC at 1- and 2-weeks post KT on clinical outcomes yielded significant associations between the occurrence of SC at 1-week post KT and death with graft function (HR 1.72 [95% CI: 1.12, 2.66]) (Supplementary Appendix, Table S5). Moreover, death with graft function was associated with Clavien Grade 1 SC at both 1-week (HR 1.76 [95% CI: 1.10, 2.83]) and 2-weeks (HR 1.77 [95% CI: 1.10, 2.84]) post KT. Readmissions were significantly associated with Clavien Grade 1 SC at 2-weeks post KT (HR 1.36 [95% CI: 1.01, 1.82]), and with Clavien Grades 3–4 at both 1-week (HR 2.51 [95% CI: 1.87, 3.36]) and 2-weeks post KT (HR 2.55 [95% CI: 1.88, 3.47]).

## Discussion

This study is one of the first to collectively investigate the incidence and severity of the full spectrum of early postoperative SC after KT, including an assessment of the impact of SC on clinical outcomes. The incidence of specific SC in our cohort is comparable to those reported by other groups [4, 15, 17]. However, this study is novel in having classified multiple categories of post-KT SC by severity and in a standardized manner, using the recently modified Clavien grading system [2]. This approach ranks the severity of SC according to their required treatment but includes subclasses to allow for more accurate capture of the clinical aftermath of SC, enhancing generalizability and interpretation of the results.

Incorporating the Clavien Grades system into our analyses allowed for a more comprehensive representation of the associations between SC and clinical outcomes. For instance, while multivariable analyses showed no relationship between the presence of SC and any outcome variables, analyzing SC by Clavien Grades revealed significant relationships between Grade 1 SC and death with graft function and between Grades 3 to 4 SC and hospital readmissions. Although death with graft function was not found to be associated with the more severe Grades 3 to 4 SC, this finding may be explained by our study exclusion criteria. In our study, 10 cases of SC among patients who experienced death or graft failure within one 30 days of transplant were excluded from outcomes analyses. One of these patients had a Grade 3 complication, and four had Grade 4 SC. Since the total number of patients with Grades 3 to 4 SC was more limited than patients with Grades 1 to 2, the severe SC are not accounted for in the mortality analyses. These 10 cases were excluded for the outcome analysis, as these patients experienced the clinical outcomes of death or graft failure within 30 days of transplant – meaning the outcome occurred before (or at the same time as) the exposure (defined as SC within 30 days of transplant).

Notably, we did find that those patients with severe SC who survive past the first month post-transplantation were more frequently admitted to the hospital thereafter. A sensitivity analysis exploring the associations between SC at different time points post KT and clinical outcomes was performed to further examine the aforementioned results. This analysis also demonstrated significant relationships between Grades 3 to 4 SC and readmissions, at both 1- and 2 -weeks. Similar to the other analyses, the less severe Grade 1 SC at 1- and 2-weeks post-KT were found to be associated with death with graft function and with hospital readmissions.

Through the utilization of the Clavien Grades system, a better understanding of exactly how SC impact clinical outcomes was achieved. Future standardization in reporting may reduce the current variation in the incidence of SC across the literature, and lead to the establishment of clinical definitions pertaining to SC. If properly defined and identified, SC are modifiable factors that can be treated by transplant care teams.

This is also one of the first studies to identify a relationship between donor type and the likelihood of having SC. When analyzed by donor type, the incidence of SC was significantly higher for ECD transplants, compared to living and non-ECD deceased donors. Only a few other studies to date have reported an increased risk of vascular complications in ECD KT [31–33]. Given the increasing number of ECD kidneys in the donor pool, this merits further investigation to ensure optimal KT outcomes.

Finally, SC represent an important quality metric and may impact patients’ overall health status. While they can occur early in the post-transplant period, SC have been shown to significantly impact hospital readmissions for up to one year post-transplant and may contribute to reduced eGFR at one year post-transplant. Moreover, the significant association between even less severe Grade 1 complications and death with graft function reinforces the role of SC as a potential quality metric.

Limitations of this study include its single centre design; however, our program is a high volume urban centre which serves a diverse patient population. Secondly, risk factors for developing SC were not explored, as they vary between different categories of SC and would be outside the scope of this project. Instead, the development of early SC itself was examined as a risk factor for adverse post-transplant clinical outcomes. We recognize that several baseline characteristics differed between patients with and without SC (Supplementary Appendix, Table S1b), warranting risk analysis in future projects. Despite the large study sample size, the number of exposed patients was considerably smaller (*N* = 329), leading to some imprecision in the effect estimates. Finally, the quality of the documentation about the presence and severity of SC varied over time, therefore, variables were systematically defined a priori and multiple personnel independently examined medical charts from electronic health records.

## Conclusions

In conclusion, early SC following KT are common and have a significant influence on long-term patient outcomes. Further studies are required to determine whether targeted strategies to specifically minimize SC are capable of improving long-term outcomes after KT.

### Supplementary Information

Supplementary Material 1.

## Data Availability

The datasets generated and/or analysed during the current study are not publicly available due the patient-sensitive nature of the data, but are available from the corresponding author on reasonable request.

## Abbreviations

eGFR: Estimated glomerular filtration rate
ECD: Expanded criteria donor
HR: Hazards ratio
KT: Kidney transplantation
SC: Surgical complications
