# Supplementary material for: Impact of early surgical complications on kidney transplant outcomes
Source: BMC Surg. 2024 May 27;24:165. doi: 10.1186/s12893-024-02463-7 (PMC11129490; doi:10.1186/s12893-024-02463-7)
Supplement: Supplementary file 1 — Supplementary Material 1. [file 12893_2024_2463_MOESM1_ESM.docx]

Supplementary table S1. Clavien Grades classification of graft-related surgical complications © Dindo D, Demartines N and Clavien P.A. 2004

| **Clavien Grade** | **Definition** |
| --- | --- |
| Grade 1 | Any deviation from normal, post-operative course without the need for pharmacological treatment or surgical, endoscopic, and radiological interventions.  Allowed therapeutic regiments include: antiemetics, antipyretics, analgesics, diuretics, electrolytes, and physiotherapy.  Also includes wound infections opened at the bedside. |
| Grade 2 | Requiring pharmacological treatment with drugs other than such allowed for Grade 1. Blood transfusions and total parenteral nutrition are also included. |
| Grade 3a | Surgical, endoscopic, or radiological interventions not under general anesthesia. |
| Grade 3b | Surgical, endoscopic, or radiological interventions under general anesthesia. |
| Grade 4a | Life-threatening complication: single organ dysfunction (including dialysis) |
| Grade 4b | Life-threatening complication: multi-organ dysfunction |
| Grade 5 | Death of a patient |

Supplementary table S2a. Baseline Characteristics of Study Population at Time of Transplant, by Donor Type (Deceased vs. Living)

| **Variables** | **Whole Cohort (n=1334)** | | **Any graft-related surgical complications within 30 Days Post-Transplant** | | | | | | | |
| --- | --- | --- | --- | --- | --- | --- | --- | --- | --- | --- |
|  |  | | **Yes (n=329)** | | | | **No (n=1000)** | | | |
|  | **Number of Patients (% with surgical complications)** | **Characteristics** | **Number of Patients** | **Deceased (n=504)** | **Living (n=496)** | **P-value** | **Number of patients** | **Deceased (n=182)** | **Living (n=138)** | **P-value** |
| **Recipient age at transplant (years)** | 1334 (24.7%) | 51.3 (± 13.5) | 329 (187 / 142) | 56.8 (± 11.7) | 48.8 (± 13.3) | < 0.001 | 1005 (507 / 498) | 55.1 (± 12.2) | 46.2 (± 13.5) | < 0.001 |
| **Recipient sex** |  | | | | | | | | | |
| Male | 798 (25.7%) | 60.3% | 203 (126 / 77) | 70.0% | 55.4% | 0.01 | 595 (309 / 286) | 61.0% | 57.4% | 0.26 |
| **Recipient Race** |  | | | | | | | | | |
| Non-white | 478 (26.1%) | 39.4% | 125 (89 / 36) | 53.9% | 28.6% | < 0.001 | 353 (235 / 118) | 50.5% | 25.8% | < 0.001 |
| White | 736 (22.5%) | 60.6% | 166 (76 / 90) | 46.1% | 71.4% |  | 570 (230 / 340) | 49.5% | 74.2% |  |
| **Recipient BMI (kg/m^2^)** | 1285 (24.8%) | 27.2 (± 5.6) | 319 (183 / 136) | 27.7 (± 5.8) | 26.9 (± 5.6) | 0.19 | 966 (484 / 482) | 27.3 (± 5.2) | 27.0 (± 5.8) | 0.31 |
| **Time on dialysis (years)** | 1334 (24.7%) | 3.2 (1.2, 5.6) | 329 (187 / 142) | 4.8 (3.2, 6.9) | 1.6 (0.3, 3.3) | < 0.001 | 1005 (507 / 498) | 5.0 (3.4, 7.1) | 1.1 (0.0, 2.5) | < 0.001 |
| **Peak PRA** |  | | | | | | | | | |
| = 0% | 659 (22.0%) | 49.9% | 145 (77 / 68) | 42.8% | 48.9% | 0.28 | 514 (213 / 301) | 42.1% | 60.7% | < 0.001 |
| > 0% | 662 (24.8%) | 50.1% | 174 (103 / 71) | 57.2% | 51.1% |  | 488 (293 / 195) | 57.9% | 39.3% |  |
| **Recipient History of Diabetes mellitus** |  | | | | | | | | | |
| Yes | 422 (27.7%) | 31.9% | 117 (76 / 41) | 42.2% | 29.5% | 0.02 | 305 (166 / 139) | 32.8% | 27.9% | 0.09 |
| **Donor Age at Donation (years)** | 1332 (24.7%) | 47.6 (± 14.5) | 329 (187 / 142) | 52.6 (± 14.2) | 46.2 (± 11.8) | < 0.001 | 1003 (506 / 497) | 48.8 (± 16.7) | 45.0 (± 12.0) | < 0.001 |
| **Donor Type** |  | | | | | | | | | |
| Living | 640 (22.2%) | 48.0% | N/A | | | | | | | |
| Deceased | 694 (26.9%) | 52.0% |  |  |  |  |  |  |  |  |
| **Expanded Criteria Donor** |  | | | | | | | | | |
| Yes | 249 (32.5%) | 18.7% | N/A | | | | | | | |
| **Double Kidney** |  | | | | | | | | | |
| Yes | 46 (34.8%) | 3.5% | 14 (16 / 0) | 8.9% | 0.0% | 0.001 | 30 (30 / 0) | 5.9% | 0.0% | < 0.001 |
| **Number of Renal Arteries** |  | | | | | | | | | |
| 1 | 1043 (23.9%) | 80.2% | 249 (136 / 113) | 77.3% | 83.1% | 0.20 | 794 (380 / 414) | 76.5% | 84.3% | 0.002 |
| 2 or more | 257 (24.5%) | 19.8% | 63 (39 / 22) | 22.7% | 16.6% |  | 194 (117 / 77) | 23.5% | 15.7% |  |
| **Number of Renal Veins** |  | | | | | | | | | |
| 1 | 1215 (24.3%) | 93.7% | 295 (165 / 130) | 94.3% | 95.6% | 0.61 | 920 (468 / 452) | 94.2% | 92.4% | 0.28 |
| 2 or more | 82 (19.5%) | 6.3% | 16 (10 / 6) | 5.7% | 4.4% |  | 66 (29 / 37) | 5.8% | 7.6% |  |
| **Cold Ischemic Time (hours) (deceased only)** | 644 (26.6%) | 11.0 (8.0, 15.1) | 171 | 11.9 (8.8, 16.4) | N/A | N/A | 473 | 10.8 (7.9, 14.2) | N/A | N/A |
| **Type of Induction** |  | | | | | | | | | |
| Non-depleting Agent | 350 (19.1%) | 26.2% | 67 (25 / 42) | 13.4% | 29.6% | 0.001 | 283 (89 / 194) | 17.6% | 39.0% | < 0.001 |
| Depleting Agent | 962 (25.8%) | 72.1% | 248 (152 / 96) | 81.3% | 67.6% |  | 714 (418 / 296) | 82.5% | 59.4% |  |
| No Induction | 22 (63.6%) | 1.7% | 14 (10 / 4) | 5.4% | 2.8% |  | 8 (0 / 8) | 0.0% | 1.6% |  |
| **Transplant Era** |  | | | | | | | | | |
| 2005-2009 | 537 (28/9%) | 40.3% | 155 (93 / 62) | 49.7% | 43.7% | 0.54 | 382 (180 / 202) | 35.5% | 40.6% | 0.01 |
| 2010-2012 | 386 (21.0%) | 28.9% | 81 (43 / 38) | 23.0% | 26.8% |  | 305 (144 / 161) | 28.4% | 32.3% |  |
| 2013-2015 | 411 (22.6%) | 30.8% | 93 (51 / 42) | 27.3% | 29.6% |  | 318 (183 / 135) | 36.1% | 27.1% |  |

Note: percentages in the “Number of patients (% with surgical complications) are row percentages (for example, 25.7% of males in the study population had a surgical complication. Percentages in the other columns are column percentages (for example, of those with surgical complications, 63.6% were males).

Supplementary table S2b. Baseline Characteristics of Study Population at Time of Transplant, Separated by Severity of Graft-Related Complication

| **Variables** | **Whole Cohort (n=1320)** | | **Any-graft related surgical complications within 30 Days Post-Transplant** | | | | | | | |
| --- | --- | --- | --- | --- | --- | --- | --- | --- | --- | --- |
|  |  | | **Yes – Separated by 2004 Clavien Grades (n=320)** | | | | | | **No (n=1000)** | **P-value** |
|  | **Number of Patients (% with surgical complications)** | **Characteristics** | **Number of patients** | **Grade 1 (n=135)** | **Grade 2 (n=96)** | **Grade 3 (n=82)** | **Grade 4 (n=7)** | **Grade 5 (n=0)** |  |  |
| **Recipient age at transplant (years)** | 1334 (24.7%) | 51.3 (± 13.5) | **1334** (135 / 96 / 85 / 13 / 0 / 1005) | 51.7 (± 13.0) | 54.0 (± 12.7) | 55.1 (± 13.4) | 54.3 (± 12.6) | N/A | 50.7 (± 13.6) | 0.01 |
| **Recipient sex** |  | | | | | | | | | |
| Male | 798 (25.7%) | 60.3% | **798** (84 / 54 / 54 / 8 / 0 / 598) | 66.7% | 56.3% | 67.9% | 61.5% | N/A | 59.2% | 0.25 |
| **Recipient Race** |  | | | | | | | | | |
| Non-white | 478 (26.1%) | 39.4% | **478** (52 / 34 / 33 / 6 / 0 / 353) | 41.6% | 42.5% | 43.4% | 60.0% | N/A | 38.2% | 0.49 |
| White | 736 (22.5%) | 60.6% | **736** (73 / 46 / 43 / 4 / 0 / 570) | 58.4% | 57.5% | 56.6% | 40.0% | N/A | 61.8% |  |
| **Recipient BMI (kg/m^2^)** | 1285 (24.8%) | 27.2 (± 5.6) | **1285** (130 / 95 / 82 / 12 / 0 / 966) | 26.6 (± 5.6) | 28.2 (± 5.8) | 27.5 (± 5.9) | 27.8 (± 5.0) | N/A | 27.1 (± 5.5) | 0.16 |
| **Time on dialysis (years)** | 1334 (24.7%) | 3.2 (1.2, 5.6) | **1334** (135 / 96 / 85 / 13 / 0 / 1005) | 3.8 (1.8, 6.0) | 3.0 (1.7, 5.5) | 3.6 (1.7, 6.2) | 3.7 (2.1, 7.4) | N/A | 3.0 (1.0, 5.5) | 0.05 |
| **Peak PRA** |  | | | | | | | | | |
| = 0% | 659 (22.0%) | 49.9% | **659** (63 / 36 / 41 / 5 / 0 / 659) | 50.0% | 37.5% | 48.8% | 38.5% | N/A | 51.3% | 0.11 |
| > 0% | 662 (24.8%) | 50.1% | **662** (63 / 60 / 43 / 8 / 0 / 488) | 50.0% | 62.5% | 51.2% | 61.5% | N/A | 48.7% |  |
| **Recipient History of Diabetes mellitus** |  | | | | | | | | | |
| Yes | 422 (27.7%) | 31.9% | **422** (42 / 39 / 28 / 8 / 0 / 305) | 33.3% | 40.6% | 33.3% | 61.5% | N/A | 30.4% | 0.05 |
| **Donor Age at Donation (years)** | 1332 (24.7%) | 47.6 (± 14.5) | **1332** (135 / 96 / 85 / 13 / 0 / 1003) | 48.3 (± 13.8) | 51.6 (± 13.2) | 49.5 (± 13.6) | 54.9 (± 13.0) | N/A | 46.9 (± 14.7) | 0.01 |
| **Donor Type** |  | | | | | | | | | |
| Living | 640 (22.2%) | 48.0% | **640** (63 / 40 / 33 / 6 / 0 / 498) | 46.7% | 41.7% | 38.8% | 46.2% | N/A | 49.6% | 0.24 |
| Deceased | 694 (26.9%) | 52.0% | **694** (72 / 56/ 52 / 7 / 0 / 507) | 53.3% | 58.3% | 61.2% | 53.9% | N/A | 50.5% |  |
| **Expanded Donor Criteria** |  |  |  |  |  |  |  |  |  |  |
| Yes | 249 (32.5%) | 18.7% | **249** (33 / 26 / 20 / 2/ 0/ 168) | 24.4% | 27.1% | 23.5% | 15.4% | N/A | 16.7% | 0.002 |
| **Double Kidney** |  | | | | | | | | | |
| Yes | 46 (34.8% | 3.5% | **46** (6 / 6 / 3 / 1 / 0 / 30) | 4.8% | 6.3% | 3.6% | 7.7% | N/A | 3.0% | 0.22 |
| **Number of Renal Arteries** |  | | | | | | | | | |
| 1 | 1043 (23.9%) | 80.2% | **1043** (102 / 67 / 72 / 8 / 0 / 794) | 82.9% | 72.8% | 85.7% | 61.5% | N/A | 80.4% | 0.09 |
| 2 or more | 257 (24.5%) | 19.8% | **257** (21 / 25 / 12 / 5 / 0 / 194) | 17.1% | 27.2% | 14.3% | 38.5% | N/A | 19.6% |  |
| **Number of Renal Veins** |  | | | | | | | | | |
| 1 | 1215 (24.3%) | 93.7% | **1215** (116 / 90 / 76 / 13 / 0 / 920) | 95.1% | 96.8% | 91.6% | 100.0% | N/A | 93.3% | 0.56 |
| 2 or more | 82 (19.5%) | 6.3% | **82** (6 / 3 / 7 / 0 / 0 / 66) | 4.9% | 3.2% | 8.4% | 0.0% | N/A | 6.7% |  |
| **Cold Ischemic Time (hours) (deceased only)** | 644 (26.6%) | 11.0 (8.0, 15.1) | **644** (66 / 50 / 48 / 7 / 0 / 473) | 12.3 (9.3, 16.2) | 11.4 (7.6, 13.8) | 14.5 (9.1, 17.7) | 10.7 (5.0, 18.0) | N/A | 10.8 (7.9, 14.2) | 0.03 |
| **Type of Induction** |  | | | | | | | | | |
| Non-depleting Agent | 350 (19.1%) | 26.2% | **350** (29 / 19 / 19 / 0 / 0 / 283) | 21.5% | 19.8% | 22.4% | 0.0% | N/A | 28.2% | < 0.001 |
| Depleting Agent | 962 (25.8%) | 72.1% | **962** (96 / 76 / 63 / 13 / 0 / 714) | 71.1% | 79.2% | 74.1% | 100.0% | N/A | 71.0% |  |
| No Induction | 22 (63.6%) | 1.7% | **22** (10 / 1 / 3 / 0 / 0 / 8) | 7.4% | 1.0% | 3.5% | 0.0% | N/A | 0.8% |  |
| **Transplant Era** |  | | | | | | | | | |
| 2005-2009 | 537 (28.9%) | 40.3% | **537** (92 / 24 / 36 / 3 / 0 / 382) | 68.2% | 25.0% | 42.4% | 23.1% | N/A | 38.0% | < 0.001 |
| 2010-2012 | 386 (21.0%) | 28.9% | **386** (25 / 28 / 23 / 5 / 0 / 305) | 18.5% | 29.2% | 27.1% | 38.5% | N/A | 30.4% |  |
| 2013-2015 | 411 (22.6%) | 30.8% | **411** (18 / 44 / 26 / 5 / 0 / 318) | 13.3% | 45.8% | 30.6% | 38.5% | N/A | 31.6% |  |

Note: percentages in the “Number of patients (% with surgical complications) are row percentages (for example, 25.7% of males in the study population had a surgical complication. Percentages in the other columns are column percentages (for example, of those with surgical complications, 63.6% were males).

Supplementary table S3. Percentage missing of the study population’s baseline characteristics at time of transplant.

| **Variable** | **Number of patients with missing characteristic (N = 1334)** | **Percent Missing** |
| --- | --- | --- |
| Recipient age at transplant (years) | 0 | 0 |
| Recipient sex | 10 | 0.75 |
| Recipient race | 120 | 9 |
| Recipient BMI (kg/m^2^) | 49 | 3.67 |
| Time on dialysis prior to transplant (years) | 0 | 0 |
| Peak PRA | 13 | 0.97 |
| Recipient history of diabetes mellitus | 11 | 0.82 |
| Donor age at donation (years) | 2 | 0.15 |
| Donor type | 0 | 0 |
| Expanded criteria donors | 0 | 0 |
| Donor history of hypertension | 39 | 2.92 |
| CVA as cause of death (Deceased only) | 13 | 0.97 |
| Donor creatinine at donoation | 22 | 1.65 |
| Donation after cardiac death (DCD) | 0 | 0 |
| Double kidney | 10 | 0.75 |
| Number of renal arteries | 34 | 2.55 |
| Number of renal veins | 37 | 2.77 |
| Type of nephrectomy regarding to donor | 14 | 1.05 |
| Side of kidney | 11 | 0.82 |
| Cold ischemic time (hours) (Deceased only) | 50 | 3.75 |
| Type of induction at transplant | 0 | 0 |
| Transplant era | 0 | 0 |

Supplementary figure S1. Cumulative Probability of: a) First Surgical Complication Over the First 30 Days After Transplant and b) First Surgical Complication Within 30 days Post-Transplant, by Donor Type


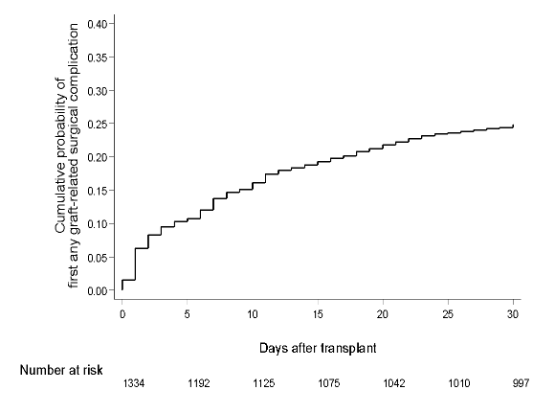

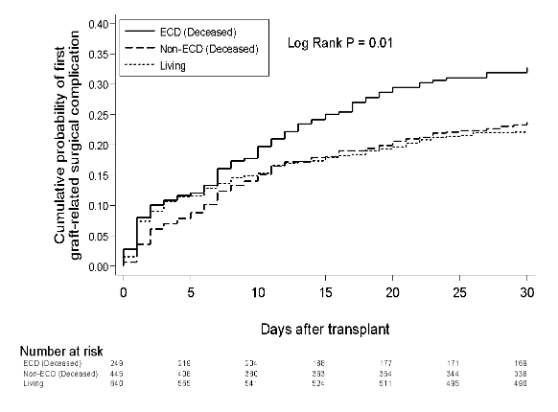


**A**

**B**

Supplementary figure S2. Incidence proportions of surgical complications, broken down by year.

Supplementary table S4. SC Cases Excluded from Outcome Analysis, with Clinical Outcomes that Occurred within 30 Days Post-Transplant

| **Patient Number** | **Transplant Date** | **Clinical Outcome** | **Date of Clinical Outcome** | **Date of SC** |
| --- | --- | --- | --- | --- |
| 1 | 10-Sep-13 | Graft Failure | 11-Sep-2013 | 11-Sep-2013 |
| 2 | 23-Jul-15 | Death | 28-Jul-15 | 24-Jul-15 |
| 3 | 9-Aug-11 | Graft Failure | 18-Aug-11 | 18-Aug-11 |
| 3* | 9-Aug-11 | Graft Failure | 18-Aug-11 | 17-Aug-11 |
| 4 | 17-Oct-14 | Graft Failure | 19-Oct-14 | 19-Oct-14 |
| 5 | 16-Aug-11 | Graft Failure | 23-Aug-11 | 30-Aug-11 |
| 5** | 16-Aug-11 | Graft Failure | 23-Aug-11 | 11-Jan-12 |
| 6 | 18-May-14 | Graft Failure | 5-Jun-14 | 18-Jun-14 |
| 6*** | 18-May-14 | Graft Failure | 5-Jun-14 | 5-Jun-14 |
| 7 | 9-Jul-09 | Graft Failure | 17-Jul-09 | 10-Jul-09 |

*Patient 3 had 2 SC

**Patient 5 had 2 SC

***Patient 6 had 2 SC

Supplementary figure S3. Association Between SC Within 30 Days Post-Transplant and Recipient eGFR over Time


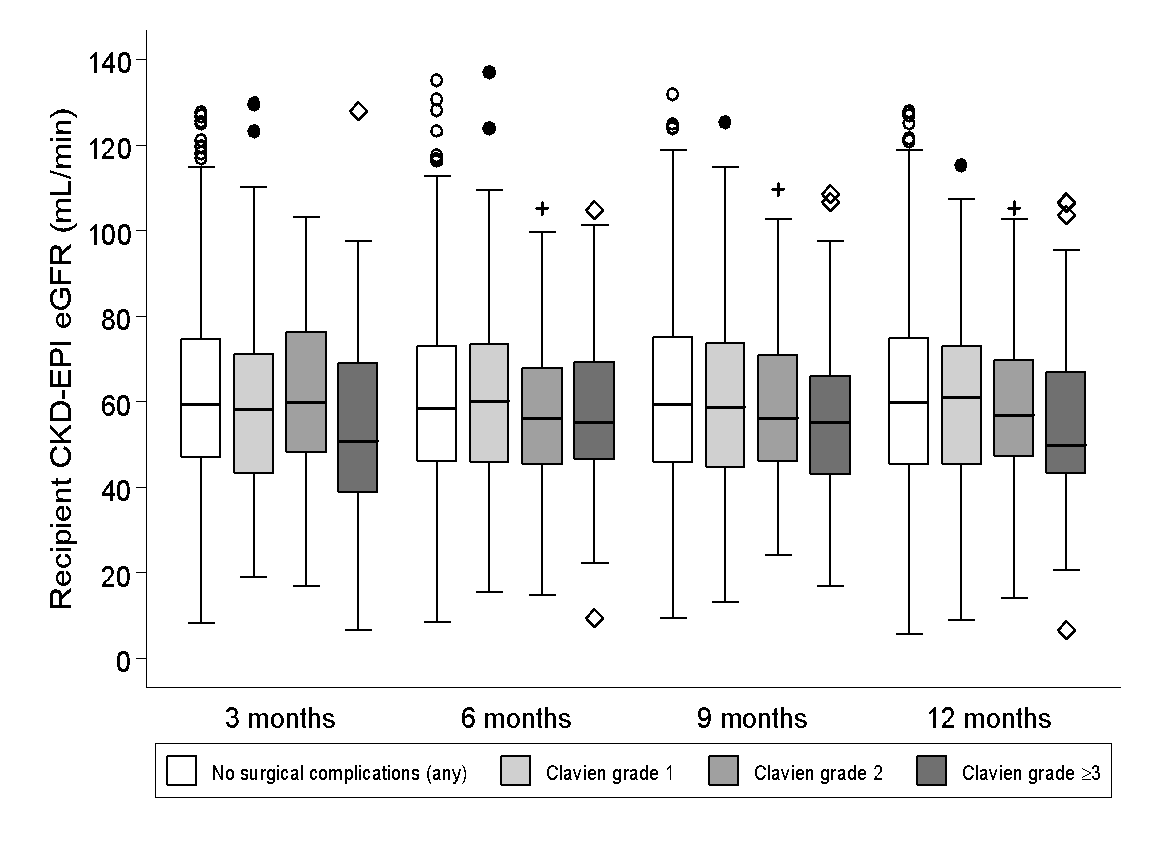


Supplementary table S5. Multivariable Cox Proportional Hazards Models for SC within 30 Days Post-Transplant and Outcomes, by Time

| **Outcomes** | **Exposure** | | **Origin: One week post-transplant (N = 1314; number of SC: 171)** | | **Origin: Two weeks post-transplant (N = 1235; number of SC: 360)** | |
| --- | --- | --- | --- | --- | --- | --- |
|  |  |  | **Hazard Ratio (95% C.I.)** | **P value** | **Hazard Ratio (95% C.I.)** | **P value** |
| **Total graft failure** | Any surgical complications | **Yes vs. No** | 1.29 (0.90, 1.84) | 0.17 | 0.96 (0.68, 1.36) | 0.83 |
|  | Clavien grades | **Grade one vs. No** | 1.28 (0.87, 1.89) | 0.21 | 1.24 (0.84, 1.84) | 0.28 |
|  |  | **Grade two vs. No** | 0.97 (0.57, 1.64) | 0.91 | 0.96 (0.56, 1.62) | 0.87 |
|  |  | **Grade three and four vs. No** | 1.47 (0.94, 2.32) | 0.10 | 1.31 (0.82, 2.10) | 0.26 |
| **Death-censored graft failure** | Any surgical complications | **Yes vs. No** | 0.77 (0.40, 1.47) | 0.43 | 0.60 (0.32, 1.12) | 0.11 |
|  | Clavien grades | **Grade one vs. No** | 0.73 (0.36, 1.47) | 0.38 | 0.64 (0.31, 1.34) | 0.24 |
|  |  | **Grade two vs. No** | 0.76 (0.30, 1.92) | 0.56 | 0.74 (0.29, 1.89) | 0.53 |
|  |  | **Grade three and four vs. No** | 2.34 (1.25, 4.39) | 0.01 | 1.95 (0.99, 3.83) | 0.05 |
| **Death with graft function** | Any surgical complications | **Yes vs. No** | 1.72 (1.12, 2.66) | 0.01 | 1.22 (0.80, 1.85) | 0.36 |
|  | Clavien grades | **Grade one vs. No** | 1.76 (1.10, 2.83) | 0.02 | 1.77 (1.10, 2.84) | 0.02 |
|  |  | **Grade two vs. No** | 1.17 (0.61, 2.22) | 0.64 | 1.16 (0.61, 2.21) | 0.65 |
|  |  | **Grade three and four vs. No** | 0.90 (0.46, 1.77) | 0.76 | 0.87 (0.44, 1.70) | 0.68 |
| **Re-admission within one year post-transplant** | Any surgical complications | **Yes vs. No** | 1.15 (0.89, 1.49) | 0.27 | 1.14 (0.90, 1.43) | 0.29 |
|  | Clavien grades | **Grade one vs. No** | 1.28 (0.96, 1.70) | 0.10 | 1.36 (1.01, 1.82) | 0.04 |
|  |  | **Grade two vs. No** | 1.34 (0.97, 1.86) | 0.08 | 1.02 (0.71, 1.47) | 0.91 |
|  |  | **Grade three and four vs. No** | 2.51 (1.87, 3.36) | < 0.001 | 2.55 (1.88, 3.47) | < 0.001 |
| **eGFR at one year** | Any surgical complications | **Yes vs. No** | 0.86 (-2.53, 4.25) | 0.62 | 1.10 (-1.85, 4.06) | 0.46 |
|  | Clavien grades | **Grade one vs. No** | 2.27 (-1.40, 5.94) | 0.23 | 2.22 (-1.44, 5.88) | 0.23 |
|  |  | **Grade two vs. No** | 1.38 (-3.14, 5.90) | 0.55 | 1.38 (-3.13, 5.89) | 0.55 |
|  |  | **Grade three and four vs. No** | -1.55 (-6.19, 3.09) | 0.51 | -0.80 (-5.45, 3.85) | 0.74 |
